# Beyond recovery: toward rights-based mental health care — A cluster randomized wait-list controlled trial of a recovery and rights training for mental health professionals with or without first person accounts

**DOI:** 10.3389/fpsyg.2023.1152581

**Published:** 2023-09-14

**Authors:** Francisco José Eiroa-Orosa

**Affiliations:** ^1^Section of Personality, Assessment and Psychological Treatment, Department of Clinical Psychology and Psychobiology, Faculty of Psychology, Institute of Neurosciences, University of Barcelona, Barcelona, Spain; ^2^First-Person Research Group, Veus, Catalan Federation of 1st Person Mental Health Organisations, Barcelona, Spain; ^3^Yale Program for Recovery and Community Health, Department of Psychiatry, Yale School of Medicine, Yale University, New Haven, CT, United States

**Keywords:** attitudes, beliefs, citizenship, education for mental health professionals, effectiveness, efficacy, recovery, rights-based care

## Abstract

**Introduction:**

Mental health models grounded in Recovery and Rights are driving the advancement of transformative care systems through multifaceted actions, which encompass Continuing Professional Development. The objective of this work is to evaluate a training activity developed through a participatory process that included people with lived experience of psychosocial distress, their relatives, and mental health professionals.

**Methods:**

The training focused on alternatives to diagnosis, recovery principles, rights-based care, and peer support. The evaluation followed a cluster randomized wait-list controlled design. Four hundred eighty-eight health professionals from eight care centers were randomized to three experimental conditions: a wait list control, which underwent a one-month interval between the baseline assessment and the training activity, and two experimental groups, with or without first-person accounts, which accessed the training immediately after completing the baseline assessment. The dependent variables measured at all follow-ups were beliefs and attitudes toward mental health service users’ rights. One hundred ninety-two professionals completed at least one follow-up and were included in the analyses.

**Results:**

We observed different evolutions of experimental and control groups with statistically significant differences for tolerance to coercion and total beliefs and attitudes scores. No differences were observed between the groups with or who attended training activities with or without first person accounts. Upon receiving the training activity, the control group had an evolution equivalent to the experimental groups.

**Discussion:**

The results of this evaluation project provide compelling evidence for the need to expand recovery and rights training activities to reach a larger audience of mental health professionals These training activities hold the potential to positively influence the beliefs and attitudes of mental health professionals, ultimately contributing toward a better future for individuals with lived experience of psychosocial distress.

## 1. Introduction

Unlike previous mental health reform movements such as anti-psychiatry, more confrontational with the status quo, the Recovery movement has taken a pragmatic approach toward disseminating its ideas among mental health professionals. Thus, while the efforts to integrate peer support workers into the care system have received more attention, the training of mental health professionals in Recovery principles has been another essential component of the movement. In the last 20 years, both the works that collect training contents of the recovery paradigm (Mabe et al., 2016), as well as narrative (Campbell and Gallagher, 2007; Jackson-Blott et al., 2019; Sreeram et al., 2021), rapid realist (Gee et al., 2017), and systematic reviews and meta-analyses (Eiroa-Orosa and García-Mieres, 2019) on the effect of these training activities have multiplied.

From a pedagogical perspective, these training activities encompass a range of learning objectives, including the acquisition of knowledge about the recovery model and its core concepts, as well as practical applications, critical analysis, and supervision. Similar to the Recovery model itself, the involvement of individuals with lived experience of psychosocial distress is a distinctive feature of these training activities, which has shown to offer additional benefits (Jackson-Blott et al., 2019). This participation allows staff to connect with the needs that are often overlooked by the Biomedical care model and discover alternative approaches proposed by the Recovery model. One of the primary goals of these training activities is to replace paternalistic practices focused solely on symptom relief with collaborative practices that prioritize the preferences and goals of service users, enabling them to reconstruct their life projects (Mabe et al., 2016). Additionally, going beyond the Recovery model, the enactment of the Convention on the Rights of Persons with Disabilities (CRPD; United Nations, 2006), has resulted in a proliferation of initiatives aimed at implementing rights-based mental health projects (Porsdam Mann et al., 2016). These include elements such as coercion reduction programs (Goulet et al., 2017; Gooding et al., 2020), the use of Advance Directives or Advance Decision Planning (Davidson et al., 2015) or legal alternatives to guardianships.

The extension of the Recovery model, especially in Anglo-Saxon countries and Western Europe, has consolidated and expanded the role of people with lived experience in training and awareness activities. Initially, these individuals were primarily former service users from the facilities where the activities were conducted. Their main function was to provide personal accounts of the experience of the care received. However, the expansion of mental health survivors’ and consumers’ movements, and the incorporation of peer support workers, facilitated their inclusion as trainers with pedagogical planning responsibilities and even leadership positions in projects aimed at implementing large-scale training activities. The scope of these activities has been very broad. The number of professionals who have participated in the countries that pioneered the Recovery model can be counted in the hundreds of thousands, including mandatory training campaigns in various territories (e.g., Way et al., 2002; Gilburt et al., 2013; Wilrycx et al., 2015). The complexity of the activities has been increasing, going from training and awareness sessions lasting a few hours, to complex processes of large-scale organizational transformation lasting several months, such as the recent REFOCUS (Slade et al., 2015) and GetREAL (Killaspy et al., 2015) projects.

Regarding the impact of these activities, the results of several reviews (Gee et al., 2017; Jackson-Blott et al., 2019), and the meta-analysis carried out by our research group (Eiroa-Orosa and García-Mieres, 2019), illustrates that recovery training for mental health professionals has a clear influence on beliefs and attitudes, while the effect on practices is less clear and very heterogeneous. It should be borne in mind that most of the studies that have measured behavioral variables have done so in the context of large-scale projects such as those already mentioned (Killaspy et al., 2015; Slade et al., 2015). This raises the question of whether it is possible to go beyond changing beliefs and attitudes, and achieve a transformation of practices, even with sufficient investment of resources. Qualitative accounts of participants in Recovery training activities (Leamy et al., 2014; Lean et al., 2015; Bhanbhro et al., 2016) offer us information to reflect on the former question. Some studies discuss the tensions between “top-down” management-led changes and “bottom-up” or team-initiated changes. In the large-scale projects mentioned, although the intention was to initiate organizational changes from the bottom up, it became apparent that the professionals involved had serious doubts about the existence of an institutional commitment to bring about tangible changes. This connects with other concepts that had already been addressed in smaller projects, but with great involvement of the participants, such as hope and autonomy. Some of the large-scale projects attempt to systematize and implement changes that first occurred spontaneously in highly committed transformative environments. Similar to the accomplishments of other social movements, when systematizing processes from the grassroots level, accounting for the unique characteristics of each context, certain contradictions emerge. One such challenge is the difficulty of replicating the innate motivation that arises organically. This seems to occur in a context in which institutions send mixed messages. On the one hand, they allocate funds to transformation projects, but on the other, they do not provide real support for the changes to take place and be maintained.

The objective of this work is to analyse the efficacy and effectiveness of a Recovery and rights-based care training activity for mental health professionals. Considering the above mentioned aspects, this activity is part of a broader initiative to transform the Catalan mental health care system (Eiroa-Orosa and Rowe, 2017). Despite substantial resource allocation in this territory toward the transformation of its mental health care system into one grounded in Recovery principles, a significant portion of the existing practices persist in relying on paternalistic approaches, hindering the active involvement of service users and their families. Our project, aimed at fostering this transformation, encompasses various stages ranging from designing evaluation instruments to implementing training and awareness initiatives targeting service users, their families, professionals, and the general population, as well as contributing to the design of public policies.

## 2. Materials and methods

### 2.1. Design and procedure

The efficacy and effectiveness of the recovery and rights training activity was evaluated through a prospective cluster randomized wait-list controlled trial. Each cluster was a mental health center. The training activity was offered through email or at events to the managers of prospective mental health centers. Once a request to carry out a training activity materialized, the name of the center was entered into a randomization table. Each center was randomly assigned to one of three conditions: a wait list control group who waited 1 month between the baseline evaluation and the training activity and one of two experimental groups, with or without first-person accounts, which accessed the training immediately after completing the baseline evaluation.

**Figure 1:**
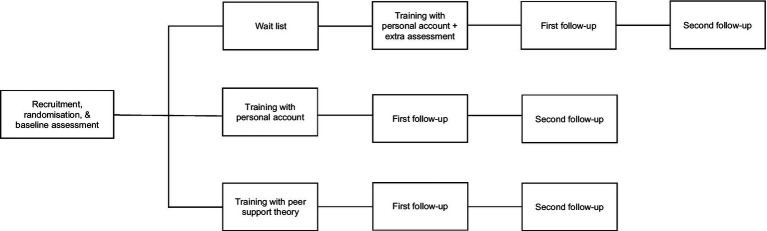
Flow diagram of the evaluation design.

Once the center was randomized, professionals received a registration questionnaire, which included the baseline assessment comprising socio-demographic, ideology, and values variables, as well as a questionnaire on beliefs and attitudes towards service users’ rights (see below). To analyse the efficacy of the training activity, the control group completed an extra evaluation just before the start of the training activity, 1 month after the baseline evaluation. All three groups received a follow-up questionnaire a week after the completion of the training activity (including satisfaction as well as the beliefs and attitudes questionnaire) and another one a month later (including the beliefs and attitudes questionnaire). Figure 1 shows a flow diagram of the evaluation procedure.

### 2.2. Participants

Considering the effect sizes of an anti-stigma intervention evaluated using a similar methodology and the same evaluation measure (Eiroa-Orosa et al., 2021a), the number of participants was estimated according to the following calculation of statistical power. Accepting an alpha risk of 0.05 and a beta risk of 0.2 in a two-sided test, assuming a correlation between the first and second measure of *r* = 0.7, 59 subjects were considered necessary in each group to recognize as statistically significant difference greater than or equal to 0.4 standard deviations.

The organizers disseminated announcements of the training activity to a wide array of healthcare providers, ensuring broad outreach. Furthermore, they took the initiative to personally present the activity during meetings of the Catalan government’s mental health advisory council, effectively engaging key stakeholders. Following acceptance of the course offer, which often involved at least one phone call or face-to-face meeting, the training activity was integrated into the Continuing Professional Development program of each center, and participants received personalized invitations that included the title of the activity (“Beyond Recovery: Toward Rights-Based Mental Health Care”), a concise overview of the learning objectives, and a direct link to the baseline questionnaire.

Following these sample size calculations, the training activity was implemented in eight mental health and primary care centers with which the desired number of participants was reached. The recipients were professionals working in such settings: administrative officers, general practitioners, nurses, psychiatrists, psychologists, social workers, etc. All centers, except for two situated in Madrid, were located within the autonomous community of Catalonia. Three centers were located within university hospitals, four within community and rehabilitation services, and one within primary care. Two centers (a university hospital and a psychosocial rehabilitation center) hosted two editions of the course.

### 2.3. Training contents

The training activity is composed of four blocks that are taught in two 4-h sessions. The contents of the activity have been elaborated through a review and meta-analysis of the literature (Eiroa-Orosa and García-Mieres, 2019), and the analysis of 20 focus groups (seven with people with lived experience, one with relatives, and 12 with professionals). For details on the entire process consult Eiroa-Orosa and Rowe (2017). With the aim of scaling this type of activities, we have published an open access handbook with versions for both trainers and trainees (Eiroa-Orosa et al., 2021b).

#### 2.3.1. First block: alternatives to diagnosis: from nosologies to shared experience

##### 2.3.1.1. Learning objectives

- Recognize preconceived ideas regarding diagnoses and nosological systems.
- Promote critical thinking toward nosological systems, and awareness of the responsibility involved in diagnosis.
- Reflect on stigma and self-stigma in mental health, emphasizing the stigmatizing attitudes of professionals, as well as the overshadowing effect of the diagnosis.
- Know the existing alternatives to nosological classifications.

##### 2.3.1.2. Contents excerpt

For centuries and up to the present day, mental health care has revolved around psychiatric diagnoses, leading to significant efforts being dedicated to the creation and refinement of nosologies such as the Diagnostic and Statistical Manual of Mental Disorders and the International Classification of Diseases. Despite the original intent to establish objectivity, research consistently reveals that the diagnostic process is far from being neutral, where solely observable symptoms are considered. Rather, there are various interfering variables that can impact the process. Among these variables we find political and religious ideologies (e.g., Gartner et al., 1990), culture and ethnicity (e.g., Bhui and Bhugra, 2002; Delphin-Rittmon et al., 2015), race, social class, or gender (e.g., Garb, 1997, 2021). On the other hand, the low reliability of certain diagnoses (Rosenhan, 1973; Regier et al., 2013) have aroused criticism of current nosological systems.

Some of the criticisms of nosological systems are structured around the prevailing biomedical conception in psychiatry. Reich (1942) was a pioneer in attaching importance to the social transmission of psychological distress, rejecting biological factors and considering socioeconomic factors as fundamental in the genesis of psychic suffering. Foucault was also critical of the Biomedical model of mental distress. According to his views, diagnosing involves establishing an arbitrary boundary between normality and pathology, which implies qualifying as pathological what is out of the ordinary (Foucault, 1954). Foucault argued that mental illness is not a timeless and universal concept, but rather a socially constructed category that has changed over time (Foucault, 1972). He claimed that the modern biomedical approach to mental illness is a form of social control that pathologizes and stigmatizes individuals who do not conform to societal norms.

The antipsychiatry movement, which involved professionals, academics and the emerging consumers and survivors movements, all critical of the provision of mental health services (Rissmiller and Rissmiller, 2006), gathered a wide range of opinions and criticisms regarding conventional psychiatric practice. Some of these criticisms are the political use of psychiatry as an instrument of social control, the medicalization of social problems, the discrimination of people diagnosed, the unresolved conflicts of interests between the psychiatric profession and the pharmaceutical industry, and involuntary and coercive treatments. All these criticisms and many others culminated in the opposition by a sector of mental health professionals to nosological systems (Beutler and Malik, 2002) especially to the fifth edition of the Diagnostic and Statistical Manual of Mental Disorders due to the poor diagnostic reliability shown in various mental disorders (Regier et al., 2013).

It is also important to highlight that receiving a mental health diagnosis has been corroborated as a predisposing factor to suffer stigma from both the social environment of the person (Schomerus et al., 2012), and by mental health professionals (Hansson et al., 2011). From a socio-cognitive perspective (Corrigan, 2006), stigma is made up of three structures: stereotypes (cognitive), prejudices (emotional), and discrimination (behavioral). Stereotypes toward people with mental health diagnoses are usually classified into three categories: dangerousness, incompetence, and permanence (Sheehan et al., 2017). This results in fear and exclusion (Corrigan et al., 2001), influencing interpersonal relationships, job opportunities, and access to housing (Stuart, 2006). Sometimes, due to the influence of the social environment, stigmatized persons themselves accept and reproduce these prejudices and discrimination toward themselves and their collective, which has been called self-stigma (Corrigan and Watson, 2002). Self-stigma can result in low self-esteem and self-efficacy (Corrigan et al., 2006), fear of stigma for encountering stigma when seeking assistance from mental health services (Rüsch et al., 2005; Thornicroft et al., 2007), reduced adherence to empirically validated treatments (Sirey et al., 2001), difficulties in recovery-oriented achievements and lower quality of life (Rüsch et al., 2010).

Alternatives to nosological systems have a long tradition (e.g., Beutler and Malik, 2002), although most proposals have not gone beyond satisfying certain therapeutic schools (Langenbucher, 2004). Due to the potentially significant impacts of diagnoses and the limited reliability of current nosological systems, it is necessary reconsider the importance of the narratives of individuals with lived experience. Actively listening to these narratives in an authentic manner, free from the biases associated with pursuing a diagnosis, can prove invaluable in uncovering resilience factors that may aid mental health service users in overcoming their challenges. Therefore, some authors (e.g., Cromby et al., 2013) have proposed systems for understanding mental suffering based on accounts of people’s experiences, as opposed to clinical nosologies. More recently, the Power Threat Meaning Framework (Johnstone and Boyle, 2018) has meant a solid formulation proposal based on the analysis of the contexts where distress and associated power imbalances occur.

#### 2.3.2. Second block: formulation and goal setting: from symptom reduction to recovery

##### 2.3.2.1. Learning objectives

- Understand the importance of formulating and establishing goals based on personal recovery and not exclusively on symptomatic relief.
- Understand the concept of Recovery, the history of the Recovery movement, its principles, and criticisms.
- Analyse how to work from the Recovery model and what can be done as professionals to promote it.
- Know the existing interventions based on the exercise of citizenship.

##### 2.3.2.2. Contents excerpt

The Recovery movement was the result of synergies between organized groups of survivors and consumers, their relatives, and mental health professionals. At the beginning of the 90s Recovery in mental health was defined as a deeply personal, unique process of changing one’s attitudes, values, feelings, goals, skills and/or roles and a way of living a satisfying, hopeful, and contributing life even with limitations caused by a mental illness (Anthony, 1993). The Recovery movement has promoted reforms of mental health care systems at various levels, especially focusing on the participation of service users and their relatives in relevant decisions (Davidson, 2016).

Some of the strategies used by mental health professionals when working from the Recovery model are: (a) the separation of the person from the diagnosis, (b) the exploration of the person’s needs, acknowledging their point of view (c) the exploration and attention to their style of autonomy, (d) the negotiation of personalized recovery plans, (e) the exploration of the power dynamics that occur between professional and service users, (f) the reduction of coercion, and (g) teamwork between services users and professionals (Davidson et al., 2016).

Although the Recovery model has made significant contributions, its implementation in some areas has drawn criticism and sparked specific protest movements such as the “Recovery in the bin movement”. These criticisms are centered on the conceptualization of a “successful recovery” that places a burden of responsibility on those who do not achieve it, the use of recovery indicators that ignore the diverse and unique nature of the concept, the individualistic application of positive psychology concepts, and the use of coercive measures such as involuntary outpatient treatment, which are disguised as “steps to recovery.”

#### 2.3.3. Third block: rights-based mental health care: collaborative practices, preferences, and advance directives

##### 2.3.3.1. Learning objectives

- Reflect on the importance of exercising rights and its relationship with the process of recovery and full citizenship.
- Know the background in the most important European and international law in the field of mental health.
- Know the United Nations convention on the rights of persons with disabilities and and its implications for people with a psychiatric diagnosis.
- Analyse the different articles of the convention, related to confidentiality, support for decision making, informed and accessible consent, health decision planning, search for alternatives to involuntary admission and the right to choose treatment.

##### 2.3.3.2. Contents excerpt

The Recovery approach is an important step in the process of improving care for mental health service users. However, it is vitally important to go one-step beyond recovery and work on the restoration of full citizenship. To achieve this, it is crucial to shift the current mental health care approach from the Biomedical model’s viewpoint, which views service users as objects of care policy, to the social model’s perspective, which sees the person as a subject of rights.

The Convention on the Rights of Persons with Disabilities included persons with lived experience of psychosocial distress in its elaboration. It recognizes rights such as equal and non-discriminatory treatment, receiving understandable information, expressing themselves freely, deciding on lives and treatment, maintaining confidentiality and privacy, receiving protection from torture and other cruel treatment, living independently, being included in the community and working (United Nations, 2006).

Despite the improvements that have occurred since the beginning of the deinstitutionalization process, currently practices considered torture and/or degrading treatment by the United Nations continue to be carried out in the field of mental health care (Gaebel et al., 2017). Examples of human rights violations include involuntary admission, forced medication, overmedication, invasion of privacy, coerced electroconvulsive therapy, mechanical restraint, confinement, and arbitrary incapacitation.

Mental health professionals have access to various tools that serve as alternatives to practices infringing upon service users’ fundamental rights. These tools include informed consent (Manning and Gaul, 1997; Roberts, 2002), advance directives and decision planning (Srebnik and La Fond, 1999; Braun et al., 2023), and peer support among individuals who have personally experienced mental health challenges (Davidson et al., 2012; Smit et al., 2022).

#### 2.3.4. Fourth block: promoting peer support: integrating the figure into the system

##### 2.3.4.1. Learning objectives

- Understand the evolution of mutual support, from its beginnings as a spontaneous phenomenon to the present day, when it is promoted as an intentional practice.
- Know the efficacy and effectiveness of mutual support, reflecting on what are the benefits of this practice.
- Analyse the ways in which the implementation of peer support into the system can be promoted.

##### 2.3.4.2. Contents excerpt

Mutual support exists spontaneously or regulated by different traditions, customs, and rituals in which the community comes together to offer support to those members who are going through difficult times. It can be said that practically any act of solidarity revolves around the idea of reciprocity, deeply rooted in all social groups. Regarding the formalization of support spaces, we could highlight one of the best-known models, the Alcoholics Anonymous mutual aid groups. Although it originated spontaneously around 1935, it was consolidated and expanded until became a standard treatment for addictions. In the field of mental health, one of the first groups that began to establish mutual aid groups was We Are Not Alone, a mental health mutual aid group established in North America in the mid-20th century.

When speaking about mutual support we can talk about three main modalities: mutual aid groups, expert patients, and professionalized peer support. The fundamental characteristic of mutual aid groups is that people can talk about their problems, achievements, and concerns as equals. They should not be confused with group therapies, since they do not include therapists, as it is not considered necessary to fulfill their objective. They are based on reciprocity and horizontality. In some groups, professionals from the mental health care network are accepted as participants, provided that they do not participate in the group as professionals, but as another member. It is also necessary to differentiate mutual support from formal expert patient programs. These consist of psychoeducational interventions taught by a person with experience of a chronic illness.

Peer support as an intentional practice (Mead et al., 2013) consists of people with lived experience who have been trained and make support their profession. It has its origins in the deinstitutionalization process and community care. With the shift toward the Recovery model, peer support has been acknowledged as a professional role within the mental healthcare field (Eiroa-Orosa and Sánchez-Moscona, 2023). While rooted in self-managed contexts, the formalization of training programs and their integration into multidisciplinary teams have enabled the recruitment and incorporation of this role into mental healthcare systems. It is essential to emphasize that the cornerstone of professionalized peer support lies in the personal experiences of the supporter and their peers. In other words, this type of support does not necessarily overlap with existing professions. The primary role of peer support is to actively listen, offer companionship, and share in the experiences of service users, providing validation and support.

Once incorporated, the tasks performed by peers can be categorized as direct or indirect support (Jacobson et al., 2012). The activities considered direct tasks are the defense of rights (providing information and support), connecting them to resources (connecting them with the desired services), sharing common experiences, building community (connecting the person with programs that link them to the community), building relationships (based on trust), facilitating group activities, developing skills and objectives, socialization, and the development of self-esteem. Indirect tasks include administration, communication, supervision, performing training, and obtaining and verifying information. In addition, the work of peer support workers also includes actions aimed at building relationships with other health professionals and legitimizing their role (Gagne et al., 2018). In this way, the tools essential for peers to fulfil their roles extend beyond their personal experiences of mental distress. They encompass life experiences of recovery and resilience, a respectful approach, genuine presence, modeling, collaboration, and active engagement (Jacobson et al., 2012).

#### 2.3.5. Instruments

Participants filled out the following questionnaires.

##### 2.3.5.1. Socio-demographic questionnaire

The first part of the questionnaire collected sociodemographic data including age, gender, qualifications, profession, years of experience, personal contact with mental disorders, and ideology (definitely left to definitely right, 5-point Likert scale).

##### 2.3.5.2. Values items adapted from the world values survey

The study incorporated two items from the World Values Survey (Inglehart, 2008) to determine where the participants were located on two continuums. The items used in the study were: “Government should ensure that everyone is provided for” (statist vs. individualist social values) and “A child needs a home with both a father and a mother in order to grow up happily” (conservative vs. progressive family values). The items had a 6-point Likert scale, ranging from “I completely disagree” to “I completely agree.”

##### 2.3.5.3. Beliefs and attitudes toward mental health service users’ rights scale

The BAMHS (Eiroa-Orosa and Limiñana-Bravo, 2019) was designed to measure mental health professionals’ beliefs and attitudes toward mental health service users’ rights. The instrument is a 25-item scale with a 4-point Likert type scale for each item. The structure of the instrument consists of four dimensions. The first subscale, Justification beliefs, pertains to mental health professionals’ beliefs that maintain the current status quo. It includes items affirming that mental disorders are diseases like any other, that aggressiveness is caused by mental disorders, that it is not possible to recover without the intervention of a professional, or that some patients will never recover. The Coercion dimension examines attitudes toward involuntary admission and the use of mechanical restraints, as well as the respect for service users’ autonomy. The Paternalism subscale reflects a set of beliefs that assume people diagnosed with mental disorders lack the capacity to manage their lives, including making decisions about having children, their treatment, or prioritizing treatment effectiveness over dignity. Finally, the Discrimination subscale embodies widespread prejudices toward mental health service users such as the ability to vote, the overuse of emergency settings, feeling comfortable becoming friends with, or feeling comfortable if a person with a mental disorder were a teacher in a school. Higher scores mean higher violation of rights. In our study reliabilities were high for the whole scale (*α* = 0.830 at baseline among participants) and moderate for the subscales (system criticism/justifying beliefs *α* = 0.658, freedom/coercion *α* = 0.510, empowerment/paternalism *α* = 0.652, and tolerance/discrimination *α* = 0.615).

##### 2.3.5.4. Satisfaction with the training activity

Together with the BAMHs, the participants filled out a satisfaction questionnaire at the first follow-up. The questionnaire consisted of items related to various aspects of the activity, including the organization, teaching methodology and style, teacher knowledge and suitability, interest of the topics, practical applications, time allocation, and materials used. The instrument showed excellent reliability (*α* = 0.913).

### 2.4. Statistical analyses

Instrument reliability was measured using Cronbach’s alpha. Attrition and differences between groups at baseline and different follow up points (including sociodemographic data and scale scores) was assessed using χ^2^ tests for categorical data and *t*-tests and analyses of variance (ANOVAs) for continuous data. Attrition was analyzed comparing baseline characteristics and scores between participants who completed the first and second follow-ups and those who did not. Ideology and values were correlated with BAMHS scores at baseline using Pearson’s r correlation coefficient. Longitudinal analyses were carried out using repeated measures general lineal models. First, to analyse efficacy, we carried out longitudinal models considering baseline scores, the extra assessment for the wait list group and the first follow-up for the experimental groups. Second, to analyse compared effectiveness, we carried out longitudinal models considering baseline scores, and both follow ups (i.e., ignoring the extra assessment of the wait list group). To account for potential confounding variables, we included ideology and values as covariates in our analyses. Additionally, we incorporated satisfaction with the training activity as a covariate specifically for the effectiveness analysis, as it was assessed at a later stage. Given that missing values were observed in the second follow-up assessment, and no statistically significant differences were found between the participants who completed the follow-up assessments and those who did not, we decided to utilize the expectation-maximization (EM) method to impute missing values and carry out intent-to-treat (ITT) analyses.

## 3. Results

**Figure 2:**
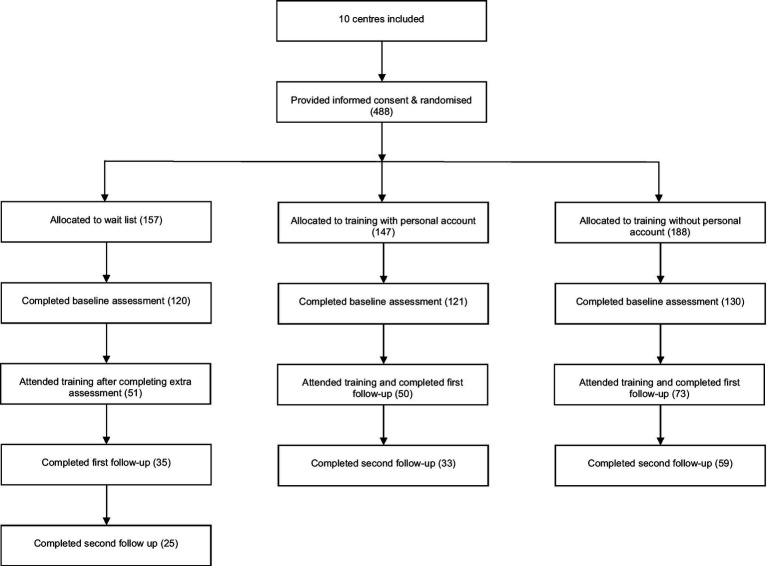
Flow the recruitment and follow-up process.

Four hundred eighty-eight health professionals from 10 healthcare centers were randomized to one of the three experimental conditions. One hundred ninety-two professionals completed at least one follow-up and were included in the analyses. Figure 2 shows a flow diagram of the recruitment and follow-up process.

Table 1 shows the baseline characteristics and scores of the three groups. No statistically significant differences were found for any variable. Similarly, no statistically significant differences were found between participants who completed the first and second follow-ups and those who did not.

**Table 1:** Sociodemographic and baseline scores of participants included in the analyses^a^ by group.

	Wait list (*n* = 64)		Training with account (*n* = 51)		Training without account (*n* = 77)				
	*n*	%	*n*	%	*n*	%	*χ*2	*p*	
Female gender	45	70.3	43	84.3	53	72.6	3.333	0.189	
Psychiatrists and psychologists^b^	27	42.2	15	29.4	25	32.9	2.299	0.317	
	***M***	**SD**	***M***	**SD**	***M***	**SD**	***F***	***p***	***η*** ^**2**^
Age	39.17	10.92	40.37	8.51	38.51	8.98	0.566	0.569	0.006
Ideology (left 1–5 right)	2.14	0.85	1.96	0.87	1.88	0.74	1.699	0.186	0.018
**Inglehart’s items**									
Statist social values	5.08	1.40	5.25	0.87	5.25	1.01	0.488	0.615	0.005
Conservative family values	2.41	1.28	2.47	1.47	2.39	1.06	0.062	0.940	0.001
**BAMHS**									
Beliefs	2.21	0.46	2.24	0.47	2.32	0.38	1.211	0.300	0.013
Coercion	2.36	0.46	2.17	0.43	2.27	0.39	2.748	0.067	0.029
Paternalism	2.09	0.43	2.17	0.52	2.25	0.37	2.234	0.110	0.024
Discrimination	1.83	0.46	1.77	0.62	1.78	0.43	0.251	0.778	0.003
Total	2.14	0.37	2.13	0.40	2.20	0.28	0.872	0.420	0.009

a To be included in the analyses, participants should have attended the training activities and completed the first follow-up.

b We dichotomised professional data to facilitate understanding, the two categories are psychiatrists and psychologists vs. nurses, social and support professionals.

Table 2 shows correlations of BAMHs scores with ideology, values and satisfaction with the training activity. All BAMHs scores were statistically significantly and moderately correlated with ideology (respect of rights with left wing). Statist vs. individualist social values only correlated negatively with Discrimination. Finally, conservative vs. progressive family values were statistically correlated with BAMHs total score and all subscales except Coercion (respect of rights with non-conservative family values).

**Table 2:** Pearson correlations of BAMHS scores with ideology and values variables.

	Ideology (left – right)	Statist social values	Conservative family values	Satisfaction with the activity
**BAMHS items**				
Beliefs	0.225**	−0.047	0.185*	−0,097
Coercion	0.355***	−0.132	0.105	−0.215**
Paternalism	0.224**	−0.055	0.318***	−0.153
Discrimination	0.266***	−0.191*	0.263***	−0.240**
Total	0.332***	−0.118	0.290***	−0.212**

*p < 0.05, **p < 0.01, ***p < 0.001.

Table 3 shows the evolution of scores across all follow-up assessments. Notably, a statistically significant difference was observed only in the coercion domain when comparing the additional assessment of the waitlist group with the first follow-up assessment of the experimental groups. *Post hoc* analysis showed that the difference existed between the control group and the treatment groups, and not between the two treatment groups. When considering the interaction of experimental group membership with evolution between these two time points, statistically significant interactions and mild effect sizes were found for coercion and the total BAMHS scores. *Post hoc* analyses suggest that the experimental groups had higher reductions in scores compared to the control group. This was confirmed by carrying out covariance analyses (ANCOVAs) in which ideology and values were added as possible confounding variables. None showed statistically significant interactions with the evolution of scores, yet the interaction with group membership remained unchanged.

**Table 3:** Follow up BAMHS per protocol scores by experimental group, differences between wait list control group extra assessment and experimental groups at first follow up, and time effect and time × group interaction at two (wait list extra vs. experimental first follow up) and three time points (baseline, first and second follow-ups).

	Wait list						Training with account				Training without account				Wait list extra assessment or experimental groups at FU1			Baseline, wait list extra assessment or experimental groups at FU1			Baseline, FU1 and FU2					
	Extra		FU1		FU 2		FU1		FU 2		FU1		FU 2		Group differences			Interaction time* group			Time effect			Interaction time* group		
	*M*	SD	*M*	SD	*M*	SD	*M*	SD	*M*	SD	*M*	SD	*M*	SD	*F*	*p*	*η* ^2^	*F*	*p*	*η* ^2^	*F*	*p*	*η* ^2^	*F*	*p*	*η* ^2^
Beliefs	2.19	0.52	2.13	0.51	2.25	0.57	2.06	0.46	2.11	0.46	2.18	0.41	2.14	0.39	1.457	0.236	0.017	2.568	0.080	0.030	8.178	<0.001	0.142	1.207	0.309	0.024
Coercion	2.27	0.45	2.18	0.56	2.18	0.52	1.98	0.49	1.89	0.44	1.96	0.44	1.89	0.48	7.704	<0.001	0.083	4.242	0.016	0.048	23.566	<0.001	0.323	0.819	0.514	0.016
Paternalism	2.08	0.40	2.08	0.50	2.10	0.62	2.11	0.43	2.05	0.53	2.16	0.41	2.08	0.43	0.567	0.569	0.007	1.207	0.302	0.014	7.308	<0.001	0.129	0.668	0.615	0.013
Discrimination	1.76	0.48	1.86	0.48	1.68	0.51	1.63	0.46	1.63	0.57	1.68	0.42	1.64	0.43	1.151	0.319	0.013	0.940	0.393	0.011	7.197	0.001	0.127	0.843	0.499	0.017
Total	2.10	0.38	2.08	0.42	2.09	0.44	1.99	0.36	1.97	0.40	2.05	0.33	1.99	0.35	1.253	0.288	0.014	4.753	0.010	0.054	21.665	<0.001	0.304	0.259	0.904	0.005

FU, Follow up.

When conducting effectiveness analyses through repeated measures ANOVAs considering all follow-up assessments and disregarding the additional assessment of the waitlist group, we found no statistically significant interaction between time and group. These findings indicate that the evolution of the scores was similar among all groups once they had received the training without differences between training modalities or with those who had to wait another month and complete an additional assessment. It is worth noting that all time effects were observed to be statistically significant and had large effect sizes, indicating that participants’ beliefs and attitudes toward the respect of rights were indeed strengthened. These findings were corroborated by performing EM to account for missing data.

When we carried out analyses of covariance to find possible confounders and predictors of the impact of the training activity, satisfaction scores showed a statistically significant positive covariation with the reduction of Coercion [*F*(2, 102) = 3.155, *p* = 0.047, *η*^2^ = 0.063] and conservative family values covaried negatively with the reduction of Paternalism [*F*(2, 102) = 3.862, *p* = 0.024, *η*^2^ = 0.076] and the total BAMHs score [*F*(2, 102) = 3.440, *p* = 0.036, *η*^2^ = 0.068]. When we carried ITT analyses with data imputed through EM we found the reduction of the Beliefs subscale to be predicted by left-wing Ideology [*F*(2, 174) = 3.146, *p* = 0.046, *η*^2^ = 0.037], statist social values [*F*(2, 174) = 4.443, *p* = 0.013, *η*^2^ = 0.051] and conservative values negatively [*F*(2, 174) = 7.969, *p* < 0.001, *η*^2^ = 0.088]. The evolution of coercive and discriminant attitudes was predicted by higher satisfaction with the training activity [Coercion: (*F*(2, 174) = 4.481, *p* = 0.013, *η*^2^ = 0.051), Discrimination: (*F*(2, 174) = 3.935, *p* = 0.021, *η*^2^ = 0.045)]. The BAMHs total score evolution was predicted by conservative values [*F*(2, 174) = 5.104, *p* = 0.007, *η*^2^ = 0.058] and Satisfaction [*F*(2, 174) = 3.786, *p* = 0.025, *η*^2^ = 0.044].

## 4. Discussion

Consistent with prior literature, our study demonstrates that a Recovery and Rights-based training had an impact on the beliefs and attitudes of mental health professionals who participated. This is supported by the divergent trajectory observed in the control group compared to the two intervention groups, as well as the similar trajectory of all groups following effective participation in the training. The findings indicate that the intervention was both efficacious and effective, with effect sizes comparable to those observed in other regions where similar interventions have been implemented (Eiroa-Orosa and García-Mieres, 2019).

The intervention demonstrated efficacy and effectiveness primarily in reducing beliefs and attitudes related to the tolerance of the use of coercion in mental health. These results are consistent with international literature on the effectiveness of this type of interventions (Goulet et al., 2017; Gooding et al., 2020) and are particularly noteworthy given the high level of awareness of the problem in the study’s context. Indeed, in Catalonia and Madrid, where the intervention was implemented, campaigns advocating for the reduction of coercion have been ongoing since 2016 (e.g., #0 Contenciones, n.d.).

Certain covariates were found to be predictors of increased impact of the training activity, particularly high satisfaction with training as a motivator and family conservative values as a deterrent. Satisfaction with the training was found to be associated with a reduction in discriminatory and coercive attitudes. Satisfaction is strongly related to engagement, and both have been found to be predictors of the impact of training activities. High levels of engagement and satisfaction can lead to better retention of training contents and increased application of the learned skills, ultimately resulting in a more positive impact of the training (Salas et al., 2012). In contrast, conservative family values appear to be a proxy for resistance to change. Consistent with the literature (DeLuca and Yanos, 2016), this variable showed statistically significant albeit moderate baseline correlations with justifying Beliefs, Paternalism, and Discrimination. The findings from the longitudinal covariance analyses suggest that individuals who harbor these beliefs exhibit a higher propensity for skepticism regarding the integration of broader horizontal practices within mental health care systems. However, the evidence suggesting that conservative mental health professionals may be more resistant to change, particularly when it comes to the Recovery model, is still scarce and it is most likely not a one-dimensional phenomenon. Indeed, the current literature illustrates that organizational factors constitute substantial barriers that go beyond the individual characteristics of the participants (Gee et al., 2017; Eiroa-Orosa and García-Mieres, 2019; Jackson-Blott et al., 2019; Sreeram et al., 2021). However, since in our study we did not have access to organizational information and transformative institutional support processes were not established systematically, we perceived that gathering these variables could furnish novel insights to the evidence. Further investigation is imperative to identify potential obstacles that mental health professionals might encounter in embracing Recovery-oriented approaches.

### 4.1. Limitations

The main limitations of this study are that the same team of trainers conducted all training activities, and the follow-up period was relatively brief. These limitations were a consequence of the lack of resources during the project implementation. To ensure successful implementation of this type of training activities on a large scale, it is essential to conduct scalability tests using sufficient resources. Hence, it is imperative to conduct further studies with adequate resources to support widespread implementation and long-term monitoring of the impact of these training activities.

In addition, the diverse range of professions, care facilities, and territories where the training actions were conducted may imply that this study was carried out in a context characterized by considerable variability. This variability encompasses both the adaptable nature of the training contents and the evaluation process. While we have incorporated certain variables to account for potential distinctions, such as considering differences between professionals based on diagnostic knowledge, in line with Corrigan’s TLC3 (Targeted, Local, Credible, Continuous Contact; Corrigan, 2011) criteria, it would be worthwhile to conduct and evaluate activities specifically tailored for targeted professional groups. Such an approach would allow for specific support and guidance during transformative processes.

Other limitations of this study are related to possible self-selection bias, and the failure to account for missing data from participants who only completed the baseline assessment. However, it should be noted that these limitations are inherent to the study’s context. The training was not mandatory, and participants could sign up for free and attend the activity based on their motivation and availability. Furthermore, attrition was relatively high, although comparable to similar studies (Eiroa-Orosa and García-Mieres, 2019). If we had treated as missing the data from people who enrolled but did not ultimately participate, it would have resulted in a multitude of possible scenarios that would have been difficult to manage. Therefore, we chose to calculate efficacy and effectiveness based on the scores of participants who effectively received the training. This approach provides a more realistic evaluation of the intervention’s impact under real-world conditions.

## 5. Conclusion

Training and awareness of professionals are fundamental elements for the implementation of Recovery and Rights-based mental health care models. Participation of people with lived experience, their relatives, as well as the involvement of all professionals, both dedicated to care and management, is essential for transformations to crystallize into care systems focused on achieving full citizenship despite experiencing psychosocial distress.

## Data availability statement

The trial was registered at the Open Science Framework website under the document object identifier https://dx.doi.org/10.17605/OSF.IO/9U4PK. The datasets analyzed for this study can be found there and as supplemental material.

## Ethics statement

All subjects gave a written informed consent in accordance with the Declaration of Helsinki. The trial was approved by the University of Barcelona Bioethics Committee (IRB00003099).

## Author contributions

The author confirms being the sole contributor of this work and has approved it for publication.

## Funding

This project has received funding from the European Union’s Framework Programme for Research and Innovation Horizon 2020 (2014–2020) under the Marie Sklodowska-Curie grant agreement N° 654808 and from the Spanish Ministry of Science and Innovation within the framework of the RYC2018-023850-I and PID2021-125403OA-I00 projects. The implementation of the training activities was partially funded by the city and provincial councils of Barcelona, the Catalan government, the Barcelona in Common’s “Filadora” programme and the Obertament association against stigma in mental health’s support programme for TLC3 actions.

## Conflict of interest

The author declares that the research was conducted in the absence of any commercial or financial relationships that could be construed as a potential conflict of interest.

## Publisher’s note

All claims expressed in this article are solely those of the authors and do not necessarily represent those of their affiliated organizations, or those of the publisher, the editors and the reviewers. Any product that may be evaluated in this article, or claim that may be made by its manufacturer, is not guaranteed or endorsed by the publisher.
